# Luteolin protects against cholestasis by suppressing hepatocyte apoptosis through direct PI3K binding and AKT activation

**DOI:** 10.3389/fphar.2026.1930755

**Published:** 2026-10-07

**Authors:** Xingliang Zhao, Yingchong Xu, Hongyan Liang, Peixu Ji, Siyao Li, Sipu Wang, Xiaopei Guo, Jie Zhang

**Affiliations:** 1 Department of Gastroenterology and Hepatology, Tianjin Medical University General Hospital, Tianjin, China; 2 Department of Gastroenterology, The Second Affiliated Hospital of Shandong First Medical University, Tai’an, Shandong, China; 3 Department of Emergency, Zhangjiagang First People’s Hospital, Zhangjiagang, Jiangsu, China; 4 Department of Rheumatology and Immunology, Dalian No.3 People’s Hospital, Dalian, Liaoning, China

**Keywords:** apoptosis, cholestasis, luteolin, PI3K/AKT pathway, *Siphonostegia chinensis* Benth., surface plasmon resonance

## Abstract

**Background:**

Excessive hepatocyte apoptosis triggered by toxic bile acids is the core pathological event of cholestasis, and the direct target of luteolin (Lut), the major bioactive constituent of *Siphonostegia chinensis* Benth., for this protective effect remains unclear. This study aims to elucidate how Lut suppresses hepatocyte apoptosis and ameliorates cholestatic liver injury.

**Methods:**

A murine cholestasis model was induced by α-naphthylisothiocyanate (ANIT), and the hepatocyte apoptotic pathways were validated in glycochenodeoxycholic acid (GCDCA)-treated HepG2 cells. Liver tissue pathological changes were assessed via hematoxylin-eosin (HE) histological staining. Network pharmacology paired with supplementary RNA sequencing (RNA-seq) was used to screen candidate pathways contributing to Lut’s anti-cholestatic effects. Quantitative real-time PCR, Western blotting and Terminal deoxynucleotidyl transferase dUTP nick-end labeling (TUNEL) immunostaining were jointly utilized to assess apoptotic levels in liver parenchymal cells. Additionally, surface plasmon resonance (SPR) coupled with molecular docking simulations was adopted to clarify the direct binding profile of Lut.

**Results:**

Lut treatment markedly ameliorated serum biochemical indices and liver histopathological lesions in ANIT-exposed mice. Meanwhile, oxidative stress was alleviated, bile acid transporters were restored, inflammatory cytokines declined, and hepatocyte apoptosis was suppressed. Network pharmacology combined with RNA-seq predicted that Lut alleviates cholestasis through the Phosphatidylinositol 3-kinase/Protein Kinase B (PI3K/AKT)-mediated anti-apoptotic pathway. This hypothesis was further supported by TUNEL, Western blotting, and qRT-PCR analyses, which indicated that Lut could activate the PI3K/AKT, and then suppress hepatocyte apoptosis in cholestasis. SPR analysis revealed a concentration-dependent direct interaction between Lut and PI3K with a kinetic KD of 9.036 × 10^−5^ M, and molecular docking revealed a plausible binding mode within the kinase domain. Additionally, the anti-apoptotic effect of Lut was largely abolished when PI3K was inhibited by LY294002 *in vitro*.

**Conclusion:**

This study is the first to confirm that Lut directly targets PI3K to initiate the PI3K/AKT signaling cascade, and thereby suppresses bile acid-induced hepatocyte apoptosis. These findings refine the mechanistic understanding of Lut’s hepatoprotective action from general antioxidant activity to target-specific anti-apoptotic signaling, and provide a molecular explanation for the traditional use of *S. chinensis* Benth. for the treatment of damp-heat jaundice.

## 1 Introduction

Cholestasis arises from impaired bile outflow coupled with extensive buildup of hepatotoxic bile acids within hepatic tissue, a pathological state that triggers excessive hepatocyte apoptosis and progressive hepatic damage (Trauner et al., 2017; Xiang et al., 2021). Among the retained bile acid species, hydrophobic variants including glycochenodeoxycholic acid (GCDCA) and lithocholic acid (LCA) are particularly injurious to hepatocytes. These bile acids can directly disrupt mitochondrial membranes, stimulate reactive oxygen species (ROS) overproduction, and initiate both endogenous and exogenous apoptotic cascades (Yokoda and Rodriguez, 2020; Asensio et al., 2022; Li et al., 2024). Progressive hepatocyte apoptosis, in turn, fuels a vicious cycle of portal inflammation, ductular reaction, and periportal fibrosis, ultimately driving disease progression toward biliary cirrhosis (Chaudhary et al., 2013; Levy et al., 2023). Furthermore, emerging evidence has illustrated that inhibiting hepatocyte apoptosis contributes to the recovery of liver damage in α-naphthylisothiocyanate (ANIT)-induced cholestatic rats (Santamaría et al., 2019; Liu et al., 2024; Yang et al., 2026). Therefore, strategies aimed at interrupting bile acid-induced hepatocyte apoptosis represent a rational therapeutic approach for cholestasis.

At present, ursodeoxycholic acid (UDCA) and obeticholic acid (OCA)—two classic farnesoid X receptor (FXR) agonists—have gained FDA approval for clinical management of cholestasis. Unfortunately, UDCA yields no therapeutic effect in over 40% of patients (Floreani and Mangini, 2018), and nearly 90% of individuals taking OCA develop a range of adverse events (Kowdley et al., 2018). This clinical reality underscores an unmet need for additional therapeutic agents that can complement the action of UDCA, particularly by targeting pathological processes that UDCA alone does not fully address—among which bile acid-triggered hepatocyte apoptosis figures prominently.

The Phosphatidylinositol 3_kinase/Protein Kinase B (PI3K/AKT) signaling cascade is a multifunctional pathway central to regulating hepatocyte survival, apoptosis and oxidative defense. In cholestatic conditions, massive accumulation of hydrophobic bile acids such as GCDCA serves as a critical stressor to hepatocytes. These toxic bile acids directly suppress PI3K/AKT signaling activity, disabling the endogenous protective network. Consequently, hepatocytes lose resistance to apoptosis and oxidative damage, accelerating the progression of cholestatic liver lesions (Chamulitrat et al., 2009; Chen et al., 2015; Li et al., 2022). Sustained PI3K activation and elevated AKT phosphorylation effectively alleviate bile acid-induced liver damage (Ma et al., 2015; Wang et al., 2024). Consistent with this notion, multiple studies using the ANIT mouse model have confirmed that PI3K/AKT is robustly inhibited during cholestasis. The natural flavonoid *tectorigenin* has been shown to reverse this pathway suppression, reduce hepatocyte apoptosis and ultimately ameliorate ANIT-triggered cholestatic injury (Ma et al., 2015; Cao et al., 2023; Lyu et al., 2025; Wang et al., 2026). Taken together, targeting the PI3K/AKT pathway represents a promising strategy for alleviating cholestasis and provides new directions for the development of novel therapeutic agents.

Preclinical studies have demonstrated that luteolin attenuates liver injury across multiple experimental models—including acetaminophen-induced hepatotoxicity, alcoholic liver disease, and non-alcoholic steatohepatitis—primarily through suppressing inflammatory responses, scavenging reactive oxygen species, and inhibiting hepatocyte apoptosis (Figure 1A) (Shanmugam et al., 2016; Yang et al., 2024; Cao et al., 2025). In the context of cholestasis specifically, Lut has been shown to regulate bile acid metabolism and reduce toxic bile acid accumulation (Wu et al., 2022), suggesting its therapeutic relevance to cholestatic liver injury. At the molecular level, accumulating evidence indicates that Lut can activate the PI3K/AKT signaling cascade (Wang et al., 2021; Hong et al., 2022; Chai et al., 2024), a central pro-survival pathway that governs hepatocyte resistance to apoptosis and oxidative damage. Notably, a recent drug affinity responsive target stability (DARTS) study has provided direct evidence that Lut physically binds to the PI3K protein (Sun et al., 2020), raising the possibility that PI3K may serve as a direct molecular target mediating Lut’s anti-apoptotic effects. Among the herbal sources of Lut, *Siphonostegia chinensis* Benth (Beiliujinu) is particularly noteworthy. As a classic traditional Chinese herb of the *Scrophulariaceae* family, Beiliujinu has been traditionally used in Chinese medicine for centuries and was documented in classical herbal literature such as *Lei Gong Pao Zhi Lun* (Lei, 1985). Characterized by a bitter and cold property and acting on the liver and gallbladder meridians, it is clinically used for damp-heat jaundice and hepatobiliary diseases, and is locally called “Tu Yinchen” for its prominent jaundice-relieving effect. Lut is designated as the quality control marker of *S. chinensis* in the Chinese Pharmacopoeia, and its abundant accumulation in this herb has been validated by UHPLC-Q-TOF MS chemical profiling (Zhao et al., 2026). This convergence of potent bioactivity and chemical abundancy positions Lut as an ideal candidate for investigating the pharmacological basis of Beiliujinu’s traditional hepatoprotective application. Importantly, ANIT-induced cholestatic liver injury in experimental animals is highly consistent with the traditional Chinese medicinal (TCM) damp-heat jaundice syndrome, which makes this model suitable for investigating the pharmacological basis of the herb’s traditional use.

**FIGURE 1:**
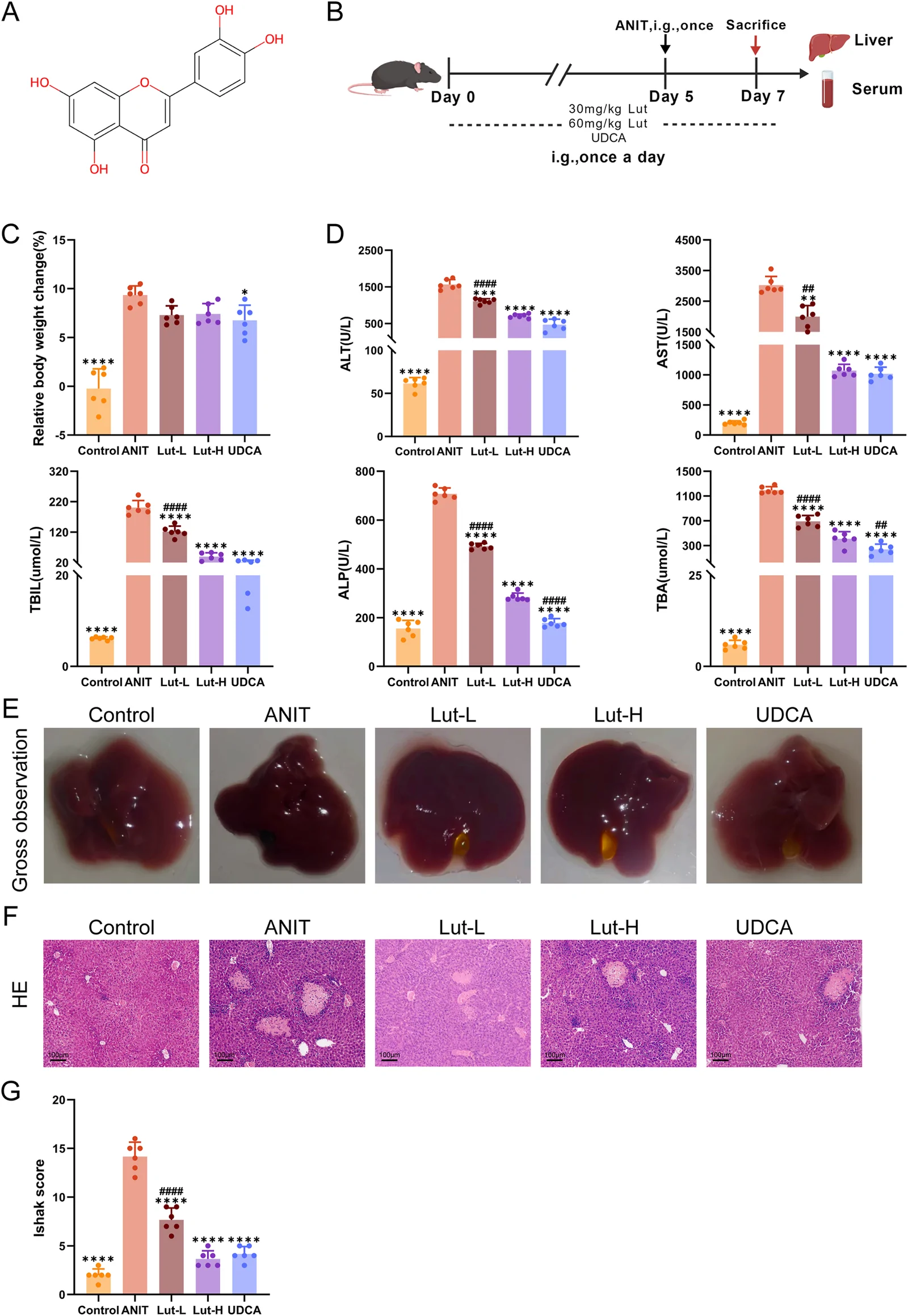
Lut ameliorates ANIT-induced liver injury in mice. **(A)** The 2D chemical structure of Lut. **(B)** Schematic diagram of the experimental design and treatment schedule. **(C)** Relative body weight change (%) of mice during the 7-day experimental period. **(D)** Serum levels of liver function markers (ALT, AST) and cholestasis markers (TBIL, ALP, TBA). **(E)** Representative gross images of livers. **(F)** Representative H&E-stained liver sections (Scale bar: 100 μm). **(G)** Histological assessment of liver injury based on the Ishak scoring system. Data are expressed as mean ± SD. *p < 0.05, **p < 0.01, ***p < 0.001, ****p < 0.0001 vs. ANIT group. ^#^ *p* < 0.05, ^##^ *p* < 0.01, ^###^ *p* < 0.001, ^####^ *p* < 0.0001 vs. Lut-H group. Only statistically significant comparisons are indicated in the figures.

However, despite the established hepatoprotective and PI3K/AKT-activating properties of Lut, a critical question remains unanswered: whether Lut directly targets PI3K to reverse bile acid-suppressed survival signaling, thereby blocking hepatocyte apoptosis during cholestatic liver injury, has not been clarified. Given the well-recognized role of bile acid-induced hepatocyte apoptosis as a key driver of cholestatic disease progression, and the absence of approved therapies that directly reinforce hepatocellular pro-survival machinery, elucidating this mechanism carries both fundamental and translational significance. This study therefore aims to determine whether Lut ameliorates ANIT-induced cholestatic liver injury through direct binding to PI3K and subsequent activation of the PI3K/AKT anti-apoptotic signaling axis, with the broader goal of providing a modern pharmacological interpretation for the traditional clinical application of *S. chinensis*.

## 2 Materials and methods

### 2.1 Network pharmacology analysis

The two-dimensional structure of Lut (CID: 5280445) was retrieved from PubChem. Putative targets of this compound were predicted based on SwissTargetPrediction (accessed on 16 March 2025), TCMSP v2.3 (accessed on 16 March 2025) (Ru et al., 2014), and CTD (accessed on 16 March 2025) (Davis et al., 2023). Meanwhile, genes associated with cholestasis were separately collected from GeneCards (accessed on 16 March 2025) (Safran et al., 2002), CTD, TTD (accessed on 16 March 2025) (Wang et al., 2020), and OMIM (accessed on 16 March 2025) (Amberger et al., 2015). All candidate gene IDs were later standardized to official human gene symbols through the UniProt database (UniProt Consortium, 2025). Venny 2.1 was adopted to screen overlapping targets between Lut and cholestasis. To dissect the functional crosstalk of these common genes, we built a human PPI network using STRING v12.0 (Szklarczyk et al., 2023), with a strict interaction confidence cutoff set at 0.9 to retain high-confidence protein pairs. The CytoNCA plugin embedded in Cytoscape v3.10.2 was used to calculate topological indicators including betweenness centrality for hub gene identification. Afterwards, Metascape (Zhou et al., 2019) was utilized to conduct GO functional annotation and KEGG pathway enrichment, and all enrichment outcomes were plotted with OmicStudio.

### 2.2 Molecular docking

The Lut 2D configuration was transformed into a 3D molecular model, and then energy minimization was done to optimize its structural stability. Then, the crystal structure of PI3K (5DXT) receptor was downloaded from the RCSB PDB database (Burley et al., 2017). PyMOL (v3.0.4) (Seeliger and de Groot, 2010) was used to remove the water molecules and original ligands, and AutoDockTools (v1.5.7) (Trott and Olson, 2010) was used to add polar hydrogen atoms and calculate Gasteiger charges. The grid box was set up according to the location of the original ligand. AutoDock (v1.5.7) (Trott and Olson, 2010) was used to do molecular docking, and the conformation with the lowest binding energy was visualized with PyMOL.

### 2.3 Surface plasmon resonance (SPR) analysis

The interaction between Lut and PI3K protein was quantitatively assessed using SPR. The PI3K protein (P27-102CH, Sino Biological, Beijing, China) was formulated into a 10 mM sodium acetate buffer (pH 4.0) and then immobilized onto the detection channel of the M5 chip. The concentration of the immobilized PI3K protein was calibrated so that the chip could reach a fixed binding capacity of roughly 10,000 RU, which is the expected response value. Lut was first prepared as a 10 mM stock solution with DMSO as the solvent, then serially diluted with operating buffer to get final concentrations from 3.125 to 10 μM. Then, these solutions were sequentially inserted into the chip for the binding-dissociation experiments. During the binding-dissociation process, the liquid flow rate was regulated to remain at 30 μL/min for the first minute, and then the subsequent dissociation period also used the same flow rate of 30 μL/min for a duration of 2 min. The SPR data were processed following channel deduction and zero concentration correction to eliminate signals attributable to flow rate and buffer effects. Data analysis was conducted using the Biacore T100 software package.

### 2.4 Reagents and antibodies

Lut (purity ≥98%, HY-N0153), ANIT (purity ≥98%, HY-B1104) and GCDCA (purity ≥97%, G275371) were purchased from Aladdin (Shanghai, China). LY294002 (HY-10108) was obtained from Beyotime (Shanghai, China). UDCA (purity ≥99%) was supplied by Dr. Falk Pharma (Freiburg, Germany). The primary antibodies used were: PI3K (bs-10657R; Bioss, Beijing, China), p-PI3K (bs-6417R; Bioss), AKT (9272S; Cell Signaling Technology, Danvers, MA, United States), p-AKT (4691S; Cell Signaling Technology), BCL2 (A11025; ABclonal, Wuhan, China), BAX (A12009; ABclonal), Caspase 3 (19677-1-AP; Proteintech, Wuhan, China), cleaved Caspase 3 (cl-Caspase 3) (25128-1-AP; Proteintech), and GAPDH (10494-1-AP; Proteintech). Horseradish peroxidase-conjugated goat anti-rabbit IgG secondary antibody (SA00001-2) was purchased from Proteintech.

### 2.5 Animal experiments

This animal study was ethically approved by Tianjin Medical University General Hospital (IRB2026-DW-84). Eight-week-old female C57BL/6 mice (18 ± 2 g) were obtained from Beijing Huafukang Biotechnology Co., Ltd. and raised in a SPF-grade environment with controlled temperature (24 °C ± 1 °C), humidity (50% ± 10%) and 12 h light-dark cycle. After 1-week acclimation, mice were randomized into five groups (n = 6 per group): control, ANIT model, 30 mg/kg low-dose Lut (Lut-L), 60 mg/kg high-dose Lut (Lut-H), and 60 mg/kg UDCA positive control group. Lut concentrations were optimized according to our preliminary data (Supplementary Figure S1). All treatments were delivered via daily intragastric administration of Lut or UDCA (dissolved in 0.5% CMC-Na) for 7 consecutive days, with control and model groups given equal volumes of 0.5% CMC-Na vehicle. On day 5, cholestasis was induced in all groups except the control via single oral administration of 60 mg/kg ANIT (dissolved in corn oil). On day 7, all mice were weighed and then euthanized by rapid cervical dislocation without prior anesthesia, in accordance with the AVMA Guidelines for the Euthanasia of Animals (2020). This procedure was performed by personnel trained and proficient in the technique. Death was confirmed by observing complete cessation of breathing and fully dilated pupils for at least 30 s. Whole blood and liver tissue samples were collected immediately following confirmation of death (Figure 1B).

### 2.6 Serum biochemical analysis

Serum was separated from clotted blood via centrifugation, and hepatic biochemical indices (ALT, AST, ALP, TBA, TBIL) were measured with an automatic biochemical analyzer and supporting kits.

### 2.7 Histopathological analysis

Liver tissues were fixed in 4% paraformaldehyde, paraffin-embedded and sectioned into 4 μm slices prior to routine hematoxylin-eosin (H&E) staining. Histopathological lesions were observed under microscopy, and necroinflammatory injury was quantified using the Ishak scoring system (Ishak et al., 1995).

### 2.8 Hepatic oxidative stress assessment

Liver oxidative stress markers were assayed with Solarbio kits per manufacturer instructions. malonaldehyde (MDA) (BC0025) was determined by TBA method at 532 nm for lipid peroxidation evaluation. superoxide dismutase (SOD) activity (BC5165) and glutathione (GSH), content (BC1175) were separately detected via WST-8 (450 nm) and DTNB (412 nm) assays to evaluate antioxidant status.

### 2.9 Apoptosis detection

Terminal deoxynucleotidyl transferase dUTP nick-end labeling (TUNEL) staining was used to detect apoptosis in liver paraffin sections and HepG2 cells with Beyotime One-Step TUNEL Kit (C1090) following manufacturer instructions. Samples were permeabilized, incubated with TUNEL reaction mixture at 37 °C, and counterstained with DAPI. Fluorescent images were captured on a KFBIO KF-FL-020 scanner (Ningbo, China). Apoptotic cell ratios were quantified with ImageJ v1.53k (NIH, Bethesda, MD, United States).

### 2.10 Real-time quantitative PCR (RT-qPCR)

Total RNA (1 μg) was isolated from liver tissues with TRIzol (15596026, Invitrogen). cDNA synthesis and qPCR were performed using ABclonal kits (RK20420, RK21200) on a QuantStudio 3 PCR instrument (Thermo Fisher). The 20 μL reaction program consisted of an initial 3 min denaturation at 95 °C, then 40 cycles of 95 °C for 5 s and 60 °C for 30 s. Primers were synthesized by Tsingke (sequences listed in Table 1). GAPDH was used as reference gene, and relative mRNA levels were quantified via the 2^−ΔΔCT^ method.

**TABLE 1:** Primers used in this study.

Target	Primer	Sequence(5′to3′)
GAPDH	Forward	TGG​CAA​AGT​GGA​GAT​TGT​TG
	Reverse	CAT​TAT​CGG​CCT​TGA​CTG​TG
PI3K	Forward	CAA​ACC​ACC​CAA​GCC​CAC​TAC​T
	Reverse	CCA​TCA​GCA​GTG​TCT​CGG​AGT​T
AKT	Forward	AGA​AGA​GAC​GAT​GGA​CTT​CCG
	Reverse	TCA​AAC​TCG​TTC​ATG​GTC​ACA​C
BCL2	Forward	ATC​GCT​CTG​TGG​ATG​ACT​GAG​TAC
	Reverse	AGA​GAC​AGC​CAG​GAG​AAA​TCA​AAC
BAX	Forward	AGA​CAG​GGG​CCT​TTT​TGC​TAC
	Reverse	AAT​TCG​CCG​GAG​ACA​CTC​G
Caspase 3	Forward	TCT​GAC​TGG​AAA​GCC​GAA​ACT​C
	Reverse	GAC​TGG​ATG​AAC​CAC​GAC​CC
BSEP	Forward	GAT​GCT​TCC​CAA​GTT​CAA​GG
	Reverse	TAA​AGA​GGA​AGG​CGA​TGA​GC
MRP2	Forward	CGA​CCA​TCC​GGA​ACG​AGT​T
	Reverse	GCA​GCC​TGT​GTG​CGA​TAG​TG
NTCP	Forward	TTC​CTG​ATG​GGC​TAC​ATT​CTC​TCT
	Reverse	GAA​TCC​TGT​TTC​CAT​GCT​GAT​G
TNF-α	Forward	AGCCGATGGGTTGTACCT
	Reverse	TGAGTTGGTCCCCCTTCT
IL-1β	Forward	GAA​GAG​CCC​ATC​CTC​TGT​GAC​T
	Reverse	GTT​GTT​CAT​CTC​GGA​GCC​TGT​AG
IL6	Forward	CTT​GGG​ACT​GAT​GCT​GGT​G
	Reverse	TCC​ACG​ATT​TCC​CAG​AGA​AC

### 2.11 Cell culture

Human hepatoma HepG2 cells (C001C001) were purchased from Beta Culture Collection (Guangzhou, China). Cells were cultured in Dulbecco’s Modified Eagle’s medium (DMEM, PM150210, Procell, Wuhan, China) supplemented with 10% fetal bovine serum (FBS, 164,210-50, Procell) and 1% penicillin-streptomycin, incubated at 37 °C under humidified 5% CO_2_.

### 2.12 Cell grouping and treatment

After seeding HepG2 cells in 6-well plates, they were randomly assigned to five groups with a total of 24 h incubation: Control group was maintained in complete medium for 24 h; GCDCA group received 12 h routine culture followed by 12 h treatment with 500 μM GCDCA; Lut group was pretreated with 15 μM Lut for 12 h before 12 h incubation with 500 μM GCDCA; LY294002 + GCDCA group was pretreated with 10 μM PI3K inhibitor LY294002 for 12 h, then exposed to 500 μM GCDCA for another 12 h; Lut + LY294002 group underwent co-pretreatment with 15 μM Lut and 10 μM LY294002 for 12 h, followed by 500 μM GCDCA treatment for 12 h.

### 2.13 Western blot analysis

Total protein was extracted using RIPA lysis buffer (R0010, Solarbio) containing 1 mM PMSF and protease/phosphatase inhibitor cocktail. Cells were lysed on ice for 30 min and centrifuged at 12,000 × g for 15 min at 4 °C. Protein concentrations were determined by BCA assay (PC0020, Solarbio). Samples were adjusted to equal concentration, mixed with 5× loading buffer, boiled at 95 °C for 5 min, and equal volumes (50 μg/lane) were separated by 10% SDS-PAGE (PI3K, p-PI3K, AKT, p-AKT, BCL2, GAPDH) or 12.5% SDS-PAGE (BAX, Caspase 3, cl-Caspase 3, GAPDH). Proteins were transferred to PVDF membranes, blocked with fast blocking buffer (BCM001, Epizyme) for 15 min at room temperature, and incubated overnight at 4 °C with primary antibodies (1:1,000). Membranes were then probed with HRP-conjugated secondary antibodies (1:5,000) for 1 h at room temperature. Bands were visualized by ECL and quantified with ImageJ, with protein expression normalized to GAPDH.

### 2.14 Statistical analysis

All statistical analyses were performed using GraphPad Prism 10.1.2 (GraphPad Software, San Diego, CA, United States). Data are expressed as mean ± standard deviation (SD). Each measured parameter was analyzed independently. Data normality was evaluated via the Shapiro–Wilk test, with P > 0.05 defined as consistent with a normal distribution. Homogeneity of variances was assessed using the Brown–Forsythe test. Our pre-specified analytical pipeline included both parametric and nonparametric workflows: for normally distributed data, ordinary one-way ANOVA with Šidák’s test (equal variance) or Welch’s ANOVA with Dunnett’s T3 test (unequal variance) was adopted; Kruskal–Wallis followed by Dunn’s multiple comparisons test was designated as the alternative for datasets with severe non-normal distribution. In the present study, a small subset of indicators (2 datasets) exhibited mild departures from normality without obvious outliers. Given the balanced sample size across all groups (n = 6 per group) and the documented robustness of one-way ANOVA to moderate violations of normality in balanced designs (Blanca et al., 2017), all datasets were analyzed using parametric ANOVA variants to maintain consistent analytical standards and facilitate unified interpretation of mean differences. Detailed test selection for each endpoint, including normality and variance homogeneity results, is summarized in Supplementary Table 2.

Statistical comparisons displayed in the main figures were based on pre-specified biological hypotheses, including comparisons between the ANIT group and each treatment group, and comparisons between the Lut-H group and the Lut-L or UDCA groups. These pre-specified primary comparisons are shown in the main figures. A complete pairwise comparison matrix covering all five experimental groups is additionally provided in Supplementary Table 3. All statistical tests were two-sided, and statistical significance was set at P < 0.05.

## 3 Results

### 3.1 Lut attenuates ANIT-induced cholestasis in mice

To evaluate the hepatoprotective capacity of Lut *in vivo*, we established an ANIT-induced cholestasis model in mice. Five groups were included: control, ANIT, ANIT with low-dose Lut (Lut-L), ANIT with high-dose Lut (Lut-H), and ANIT with UDCA. For relative body weight fluctuations (Figure 1C), the control group showed a significant difference compared with the ANIT group (*P* < 0.0001). The UDCA group also exhibited a significant difference relative to the ANIT group (*P* = 0.0153), while the Lut-L and Lut-H groups showed no significant differences compared with the ANIT group (Lut-L: *P* = 0.0764; Lut-H: *P* = 0.052).

Serum biochemical tests confirmed the successful establishment of ANIT-induced cholestasis, as serum ALT, AST, ALP, TBIL, and TBA levels were all markedly elevated in the ANIT group compared with the control group (all *P* < 0.0001) (Figure 1D). Compared with the ANIT group, Lut-L administration significantly reduced the elevated levels of ALT (*P* = 0.0007), AST (*P* = 0.002), TBIL (*P* < 0.0001), ALP (*P* < 0.0001), and TBA (*P* < 0.0001), while Lut-H treatment produced even more pronounced reductions (all *P* < 0.0001 vs. ANIT). Notably, Lut-H exerted a significantly stronger protective effect than Lut-L for all detected serum biochemical indicators (ALT: *P* < 0.0001; AST: *P* = 0.0045; TBIL: *P* < 0.0001; ALP: *P* < 0.0001; TBA: *P* < 0.0001). The efficacy of Lut-H was comparable to that of UDCA for ALT (*P* = 0.0521), AST (*P* = 0.9427), and TBIL (*P* = 0.2216), but Lut-H showed a significantly better effect on ALP (*P* < 0.0001) and TBA (*P* = 0.0062). Gross examination and H&E staining of liver tissues further confirmed that Lut ameliorated ANIT-induced hepatic necrosis, inflammatory infiltration, and bile duct proliferation (all *P* < 0.0001 vs. ANIT) (Figures 1E–G).

To delve further into the effects of Lut on parameters related to cholestasis, we examined the mRNA levels of key bile acid transporters and inflammatory cytokines in liver tissues. Compared with the ANIT group, both Lut-L and Lut-H significantly restored the expression of bile acid transporters: Lut-L upregulated the efflux transporters BSEP (*P* = 0.0024) and MRP2 (*P* = 0.0153), and downregulated the uptake transporter NTCP (*P* < 0.0001), while Lut-H produced more significant regulatory effects on all three transporters (all *P* < 0.0001 vs. ANIT). Lut-H exerted a significantly stronger regulatory effect than Lut-L on BSEP (*P* = 0.0002), MRP2 (*P* = 0.0003), and NTCP (*P* = 0.0125), indicating a dose-dependent improvement in bile acid transport function (Figure 2A).

**FIGURE 2:**
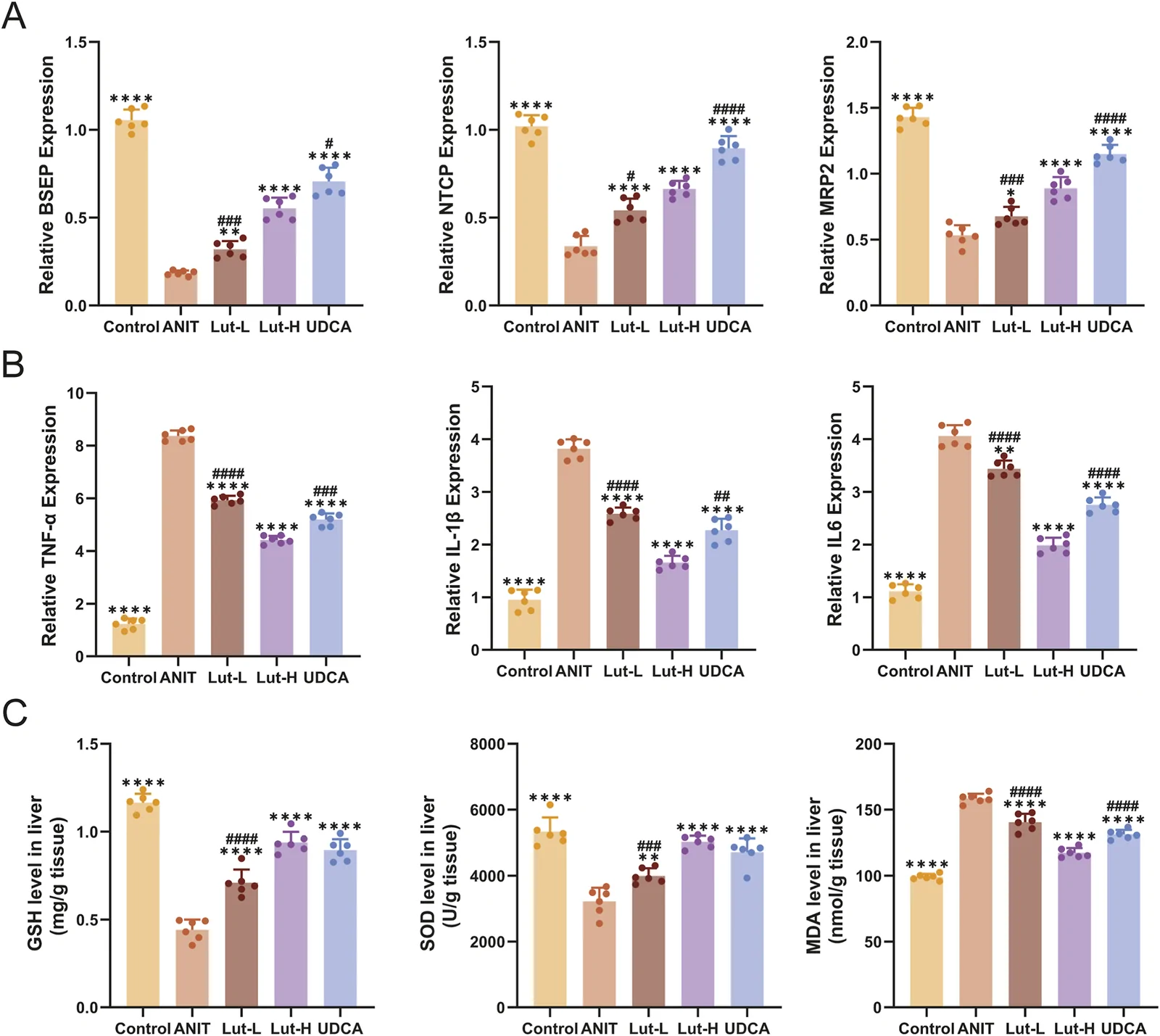
Lut modulates bile acid transporters, inflammatory cytokines, and oxidative stress markers in ANIT-induced cholestasis. **(A)** The relative mRNA expression levels of BSEP, MRP2, NTCP in liver tissues. **(B)** The relative mRNA expression levels of TNF-α, IL-1β and IL6. **(C)** Hepatic levels of glutathione (GSH), superoxide dismutase (SOD), and malonaldehyde (MDA). Data are expressed as mean ± SD. *p < 0.05, **p < 0.01, ***p < 0.001, ****p < 0.0001 vs. ANIT group. ^#^ *p* < 0.05, ^##^ *p* < 0.01, ^###^ *p* < 0.001, ^####^ *p* < 0.0001 vs. Lut-H group. Only statistically significant comparisons are indicated in the figures.

Furthermore, Lut reduced the mRNA expression of pro-inflammatory cytokines including TNF-α, IL-1β, and IL6 (all *P* < 0.01) (Figure 2B). We also analyzed the oxidative stress markers in liver homogenates. ANIT challenge markedly reduced GSH content and suppressed SOD activity, and increased MDA content (all *P* < 0.0001 vs. control). Lut treatment significantly reversed these abnormal indicators (all *P* < 0.01 vs. ANIT) (Figure 2C).

Collectively, these results indicate that Lut not only protectes hepatocytes from bile acid mediated injury, but also partially improved bile acid transport and reduced oxidative stress and inflammatory responses in the cholestatic liver and these protective effects are dose-dependent.

### 3.2 Identification of PI3K/AKT as a candidate pathway mediating the hepatoprotective effect of Lut

After confirming Lut’s effectiveness in animal models, we sought to explore the molecular pathways underlying its hepatoprotective effect. We first conducted network pharmacology analysis, which identified 142 overlapping targets between Lut and cholestasis-associated genes (Figure 3A). GO analysis results are shown in Supplementary Figure S2. KEGG pathway enrichment analysis revealed that the PI3K/AKT signaling cascade and apoptotic cascade ranked among the most significantly enriched pathways (Figure 3B and Supplementary Table). These results suggested that Lut’s hepatoprotective effect might involve modulation of PI3K/AKT-related cell survival and anti-apoptotic mechanisms. In the PPI network analysis, we found that AKT1 ranked within the top 10 core nodes by betweenness centrality (Figures 3C,D and Supplementary Table), suggesting it acted as a vital signaling hub within this network. Although PI3K itself was not among the top hub nodes, it was the main upstream activating kinase of AKT, and was prominently identified in the KEGG enrichment analysis. Therefore, we hypothesized that Lut might activate downstream AKT signaling by directly targeting PI3K, rather than acting on AKT itself.

**FIGURE 3:**
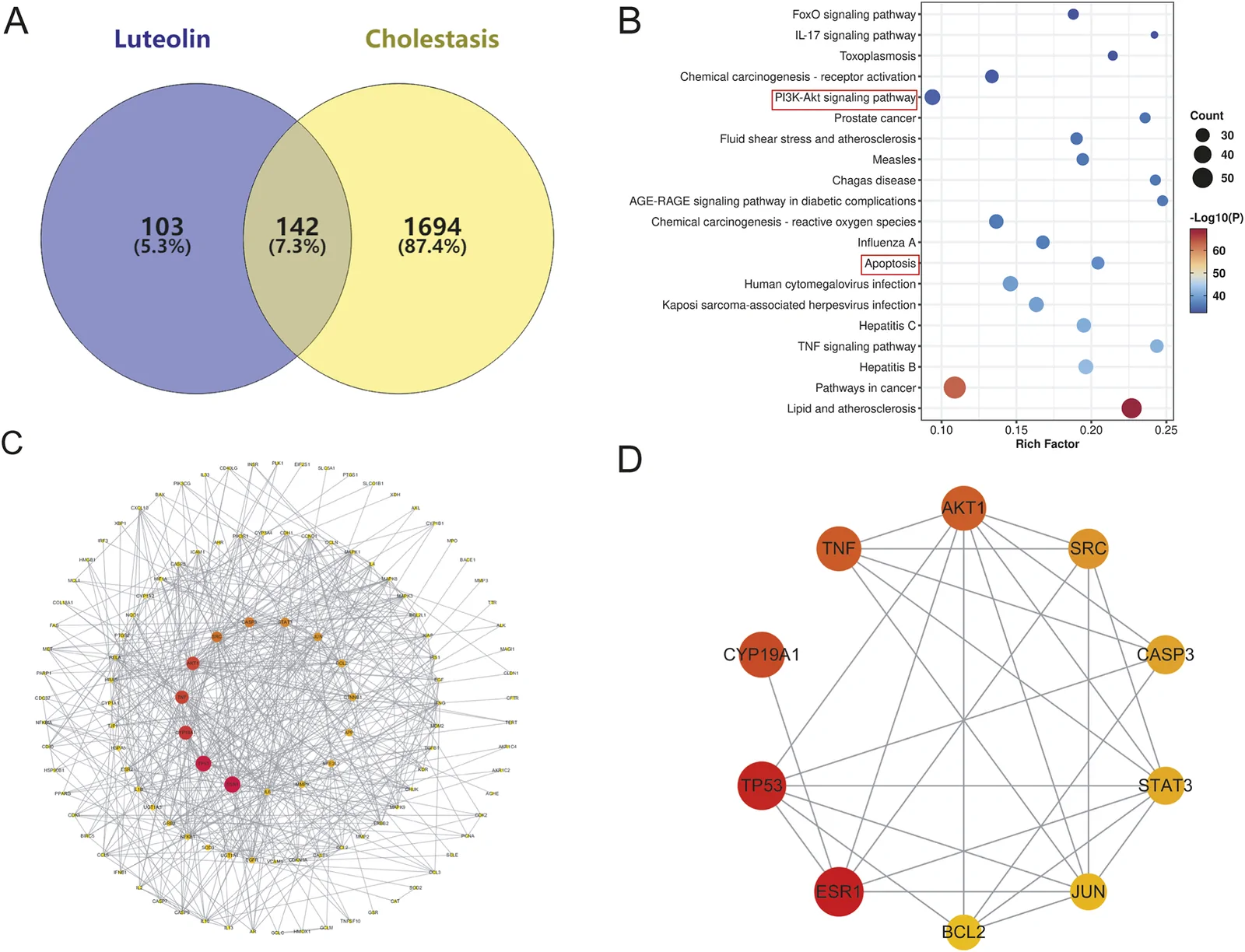
Network pharmacology predicts therapeutic mechanism of Lut against cholestasis. **(A)** Venn diagram showing 142 intersecting targets between Lut and cholestasis. **(B)** Kyoto Encyclopedia of Genes and Genomes (KEGG) pathway enrichment analysis of the intersecting targets. Bubble size indicates the number of enriched genes, and color gradient represents the significance level (*p*-value); the x-axis represents rich factor; the y-axis lists enriched pathways. **(C)** PPI network of the 142 intersecting targets. **(D)** Top 10 core targets of the PPI network.

To obtain an unbiased transcriptional perspective independent of the network pharmacology prediction, we conducted RNA sequencing on liver tissues from control, ANIT, and ANIT + Lut groups (Supplementary Figure S3. Although the modest sample size limited the statistical power for comprehensive differential expression analysis, KEGG enrichment analysis of the transcriptional changes consistently highlighted the PI3K/AKT signaling cascade as one of the most prominently altered pathways upon Lut treatment. Convergent outputs from orthogonal network pharmacology and RNA-seq analyses guided our follow-up research on this pro-survival pathway.

### 3.3 Lut directly binds to PI3K *in vitro*

To examine the potential direct interaction between Lut and PI3K, SPR experiments were used with purified recombinant PI3K protein immobilized on a sensor chip. Lut bound to PI3K in a concentration-dependent manner. Global kinetic fitting yielded a KD value of 9.036 × 10^−5^ M, while steady-state affinity analysis produced a KD value of 2.82 × 10^−4^ M (Figure 4A). Since the kinetic model incorporates the complete association and dissociation phases, the kinetic KD was employed as the primary affinity parameter in this study. The discrepancy between the two estimates may indicate incomplete saturation during the steady-state analysis and slight deviations from ideal binding behavior. To further explore the binding mode, we conducted molecular docking analysis. Lut could be placed in the ATP-binding pocket of the PI3K kinase domain, thereby forming hydrogen bonding interactions with key amino acid residues including glu135, arg683, gln682, ser464, ser681, thr679 (Figure 4B). These results suggest that Lut could directly interact with PI3K through specific molecular interactions.

**FIGURE 4:**
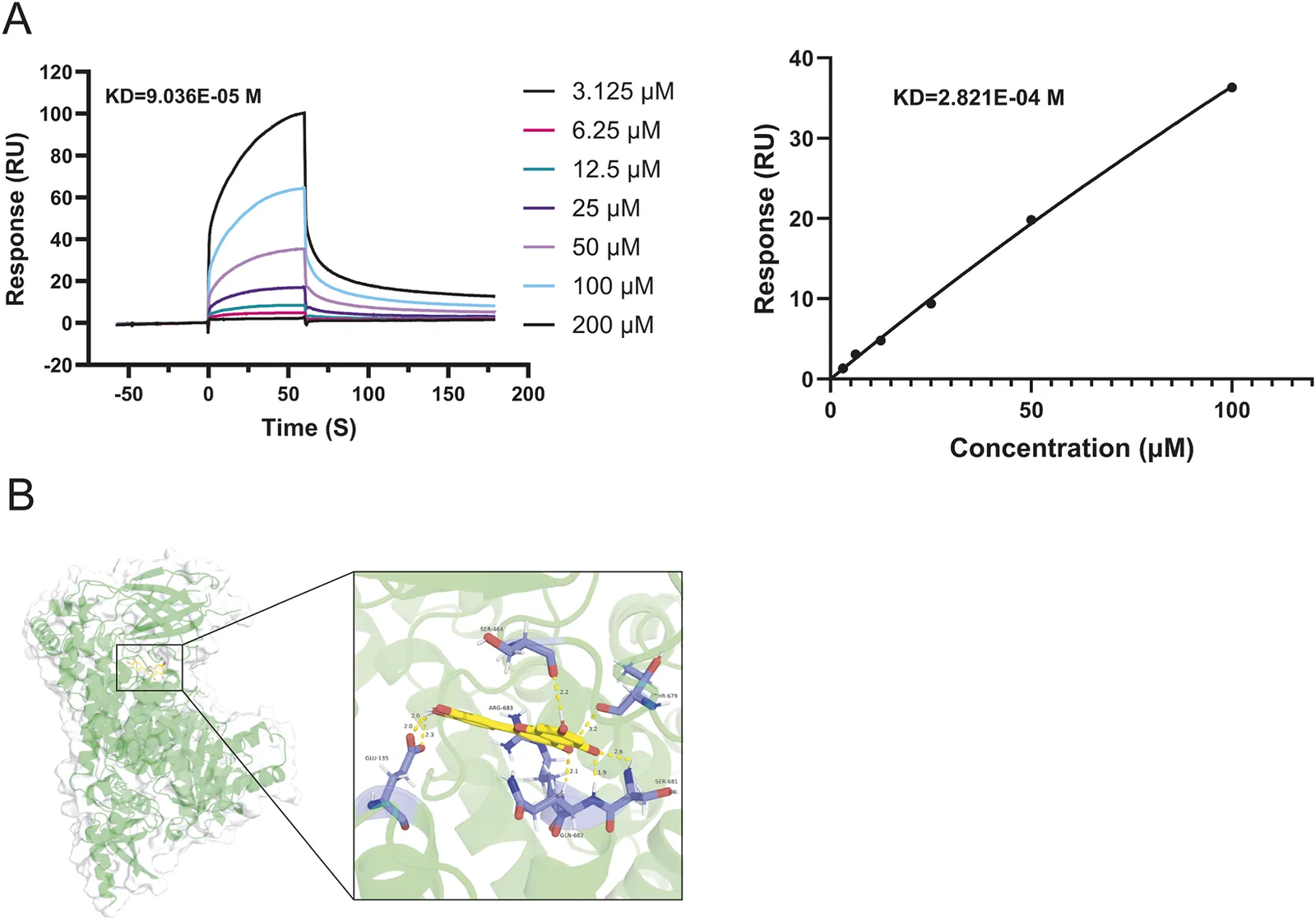
Binding analysis of Lut to PI3K via molecular docking and SPR. **(A)** The steady-state fit curve and dynamic fit curve of Lut and PI3K via SPR. **(B)** The predicted binding mode of Lut within the kinase domain of PI3K (PDB ID: 5DXT). The full-length PI3K catalytic subunit structure is shown as a green ribbon, Lut is depicted as yellow stick structures. The enlarged view shows the interaction between Lut and key residues within the ATP-binding pocket.

### 3.4 Lut activates the PI3K/AKT pathway and inhibits apoptosis in ANIT-induced cholestatic mice

We next explored whether Lut modulates the PI3K/AKT signaling cascade and downstream apoptotic events in ANIT-induced cholestatic liver injury. For all endpoints in this section, complete pairwise comparisons across the five experimental groups are provided in Supplementary Table 3.

#### 3.4.1 Transcriptional changes of the PI3K/AKT signaling pathway

qRT-PCR results demonstrated that ANIT challenge markedly suppressed the mRNA transcription of PI3K and AKT in liver tissues (*P* < 0.0001 vs. control group). Both Lut-L and Lut-H significantly rescued the downregulated PI3K and AKT transcripts relative to the ANIT group (all *P* < 0.0001). Lut-H exerted a significantly stronger restorative effect than Lut-L for both genes (PI3K: *P* < 0.0001; AKT: *P* < 0.0001), while no significant difference was observed between Lut-H and the positive control UDCA (PI3K: *P* > 0.9999; AKT: *P* = 0.8841) (Figures 5A,B).

**FIGURE 5:**
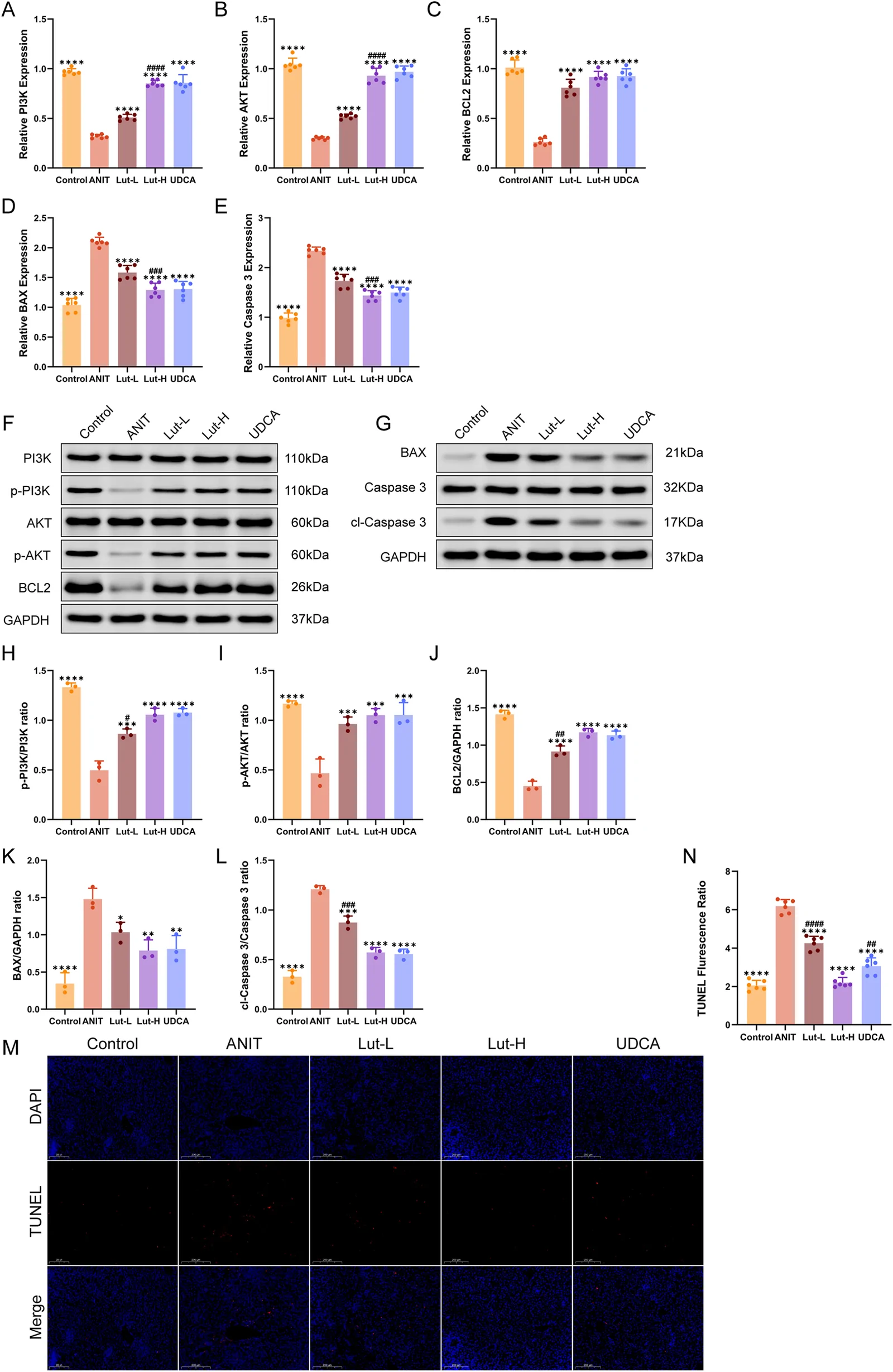
Lut activates PI3K/AKT signaling and regulates apoptosis-related factors *in vivo*. **(A–E)** The relative mRNA expression levels of PI3K **(A)**, AKT **(B)**, BCL2 **(C)**, BAX **(D)**, and Caspase 3 **(E)** in liver tissues were determined by RT-qPCR. **(F,G)** Representative Western blot images showing the protein expression of PI3K, p-PI3K, AKT, p-AKT, BCL2 **(F)**, and BAX, Caspase 3, cleaved-Caspase 3 (cl-Caspase 3) **(G)**. GAPDH was used as the loading control for normalization of each corresponding protein expression analysis. **(H-L)** Quantitative analysis of the Western blot results: p-PI3K/PI3K ratio **(H)**, p-AKT/AKT ratio **(I)**, relative BCL2 protein level **(J)**, relative BAX protein level **(K)**, and cl-Caspase 3/Caspase 3 ratio **(L)**. **(M)** Representative immunofluorescence images of TUNEL staining in liver tissues. Red fluorescence indicates TUNEL-positive apoptotic cells, and blue fluorescence indicates DAPI-stained nuclei (Scale bar: 200 μm). **(N)** Quantitative analysis of the ratio of TUNEL-positive cells. Data are expressed as mean ± SD. *p < 0.05, **p < 0.01, ***p < 0.001, ****p < 0.0001 vs. ANIT group. ^#^ *p* < 0.05, ^##^ *p* < 0.01, ^###^ *p* < 0.001, ^####^ *p* < 0.0001 vs. Lut-H group. Only statistically significant comparisons are indicated in the figures.

We further examined the transcriptional expression of apoptosis-related genes downstream of the PI3K/AKT pathway. Lut significantly upregulated the anti-apoptotic gene BCL2 and reversed the ANIT-mediated upregulation of the pro-apoptotic genes BAX and Caspase 3 compared with the ANIT group (all *P* < 0.0001). For BAX and Caspase 3, Lut-H exhibited a significantly more potent inhibitory effect than Lut-L (BAX: *P* = 0.0007; Caspase 3: *P* = 0.0003), while no significant difference in BCL2 mRNA levels was observed between Lut-L and Lut-H (*P* = 0.0667). No significant differences were detected between Lut-H and UDCA for the three apoptotic genes (BCL2: *P* > 0.9999; BAX: *P* > 0.9999; Caspase 3: *P* = 0.8982) (Figures 5C–E).

#### 3.4.2 Protein phosphorylation changes of the PI3K/AKT signaling pathway

Western blot analysis of hepatic tissue further validated these transcriptional alterations at the protein level. ANIT exposure markedly reduced the phosphorylation ratios of PI3K and AKT (p-PI3K/PI3K and p-AKT/AKT) (both *P* < 0.0001 vs. control). For the p-PI3K/PI3K ratio, both Lut-L and Lut-H significantly restored phosphorylation levels relative to the ANIT group (Lut-L: *P* = 0.0001; Lut-H: *P* < 0.0001), with Lut-H producing a significantly stronger effect than Lut-L (*P* = 0.0178), showing clear dose-dependent activation. For the p-AKT/AKT ratio, both doses also induced significant improvement versus ANIT (Lut-L: *P* = 0.0005; Lut-H: *P* = 0.0001), but no significant difference was detected between Lut-L and Lut-H (*P* = 0.8616). No significant differences were found between Lut-H and UDCA for either phosphorylation ratio (p-PI3K/PI3K: *P* = 0.9991; p-AKT/AKT: *P* > 0.9999) (Figures 5F–I). Collectively, these data confirm that Lut rescues ANIT-mediated suppression of the PI3K/AKT pathway, with partial dose-dependent effects on PI3K phosphorylation.

#### 3.4.3 Expression changes of apoptosis-related proteins

Consistent with PI3K/AKT pathway activation, Lut significantly upregulated the anti-apoptotic BCL2 protein (*P* < 0.0001 vs. ANIT), and Lut-H produced a significantly higher BCL2 expression level than Lut-L (*P* = 0.0025). Lut also alleviated ANIT-induced BAX protein accumulation: Lut-L significantly reduced BAX protein levels relative to ANIT (*P* = 0.0275), and Lut-H showed a more significant reduction (*P* = 0.0013), while no significant difference in BAX levels was detected between Lut-L and Lut-H (*P* = 0.3651). No significant differences were observed between Lut-H and UDCA for both BCL2 and BAX proteins (BCL2: *P* = 0.968; BAX: *P* > 0.9999) (Figures 5J,K). Moreover, Lut robustly decreased the ratio of cl-Caspase 3 to total Caspase 3 (both *P* < 0.001 vs. ANIT), and the suppressive effect was significantly stronger in the Lut-H group (*P* = 0.0002). No significant difference was detected between Lut-H and UDCA for this endpoint (*P* = 0.9993) (Figure 5L).

#### 3.4.4 Hepatocyte apoptosis quantification by TUNEL staining

TUNEL staining was performed to quantify hepatocyte apoptosis in liver tissue sections. The number of apoptotic hepatocytes was markedly increased in the ANIT group compared with the control group (*P* < 0.0001). Both Lut-L and Lut-H treatment markedly reduced apoptotic signals relative to the ANIT group (all *P* < 0.0001). The Lut-H group exhibited a significantly lower apoptosis ratio than the Lut-L group (*P* < 0.0001), confirming dose-dependent anti-apoptotic activity. Notably, Lut-H showed a significantly stronger inhibitory effect on hepatocyte apoptosis than the positive control UDCA (*P* = 0.0011) (Figures 5M,N).

Taken together, these results demonstrate that Lut activates the hepatic PI3K/AKT pro-survival signaling axis and subsequently suppresses the BCL2/BAX/Caspase-3 apoptotic cascade, thereby alleviating ANIT-induced cholestatic liver injury *in vivo*.

### 3.5 Lut protects HepG2 cells against GCDCA-induced apoptosis through the PI3K/AKT pathway

To further dissect the mechanistic role of the PI3K/AKT pathway in Lut’s anti-apoptotic activity, we established an *in vitro* cholestatic model by treating HepG2 cells with GCDCA, a hydrophobic bile acid that recapitulates toxic bile acid stress. The optimal GCDCA concentration was determined by CCK-8 assay, and 500 μM was selected for subsequent experiments (Supplementary Figure S4). GCDCA markedly aggravated oxidative stress, as evidenced by decreased GSH content and SOD activity accompanied by elevated MDA levels (all *P* < 0.0001 vs. control). Co-incubation with 10 μM or 15 μM Lut effectively rescued these abnormal oxidative stress markers (all *P* < 0.01 vs. control) (Figures 6A–C).

**FIGURE 6:**
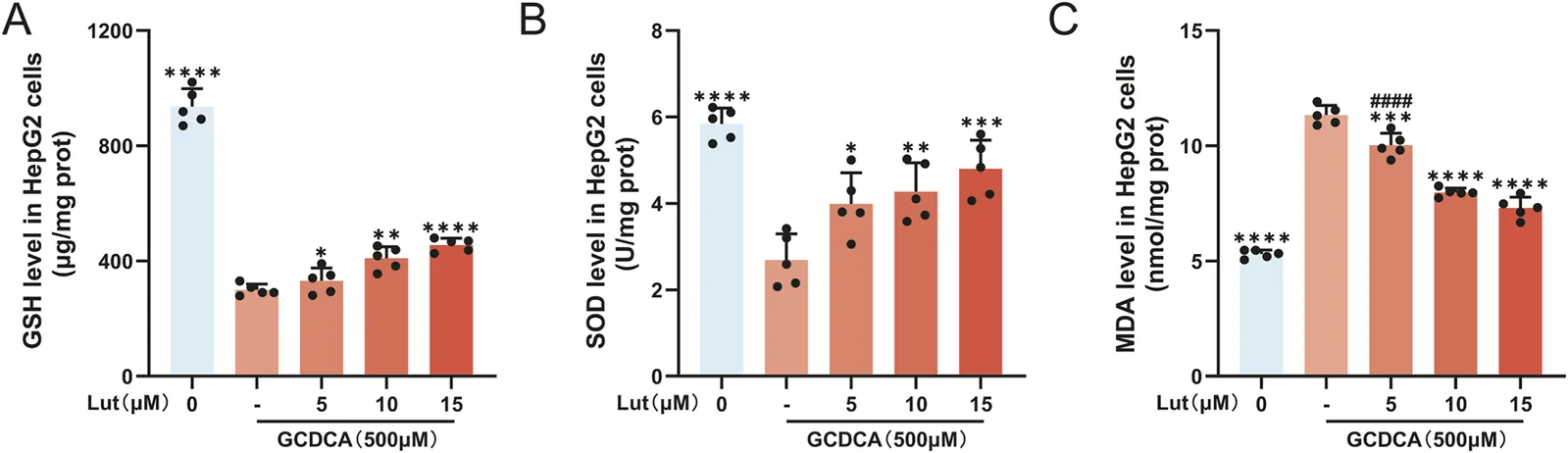
Lut alleviates HepG2 cell oxidative stress. **(A-C)** HepG2 cell levels of GSH, SOD, and MDA. Data are expressed as mean ± SD. *p < 0.05, **p < 0.01, ***p < 0.001, ****p < 0.0001 vs. GCDCA group. ^#^ *p* < 0.05, ^##^ *p* < 0.01, ^###^ *p* < 0.001, ^####^ *p* < 0.0001 vs. 10 μM Lut group.

Having confirmed that Lut alleviates GCDCA-induced oxidative stress, we next examined whether this protection involves the PI3K/AKT signaling axis, a key regulator of both antioxidant defense and apoptotic cell death. Western blot analysis revealed that GCDCA treatment markedly suppressed the phosphorylation of PI3K and AKT, decreased the BCL2/BAX ratio, and upregulated cl-Caspase 3 (all *P* < 0.0001), indicating activation of the mitochondrial apoptotic pathway (Figures 7A–G). Co-treatment with Lut effectively reversed all these changes: p-PI3K and p-AKT levels were restored, BCL2 expression was elevated, and both BAX and cl-Caspase 3 levels were significantly reduced (all *P* < 0.001). In contrast, co-administration of the PI3K inhibitor LY294002 with Lut largely prevented the recovery of p-PI3K and p-AKT, blocked BCL2 upregulation, and maintained BAX and cl-Caspase 3 at levels comparable to those in the GCDCA-treated group (all *P* < 0.05). TUNEL staining confirmed these findings: Lut decreased the proportion of TUNEL-positive apoptotic cells (12.46 ± 0.8599 vs. 120.3 ± 12.92 in GCDCA, *P* < 0.0001), and this protective effect was nearly abolished by LY294002 (12.46 ± 0.8599 vs. 69.08 ± 22.79 in GCDCA + Lut + LY294002, *P* < 0.0001) (Figures 7H,I).

**FIGURE 7:**
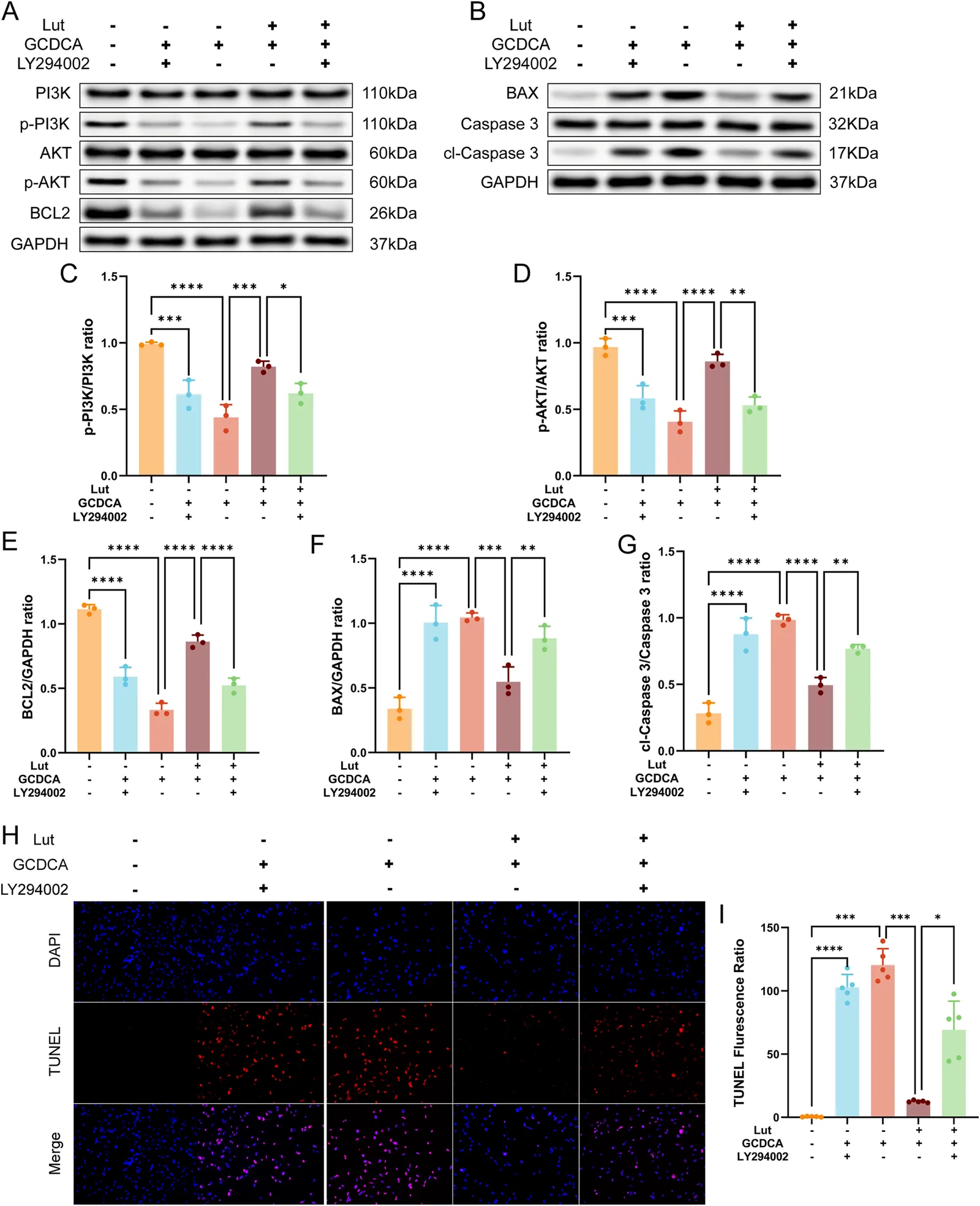
PI3K inhibition abolishes the protective effects of Lut in HepG2 cells. **(A,B)** Representative Western blot images showing the protein expression of PI3K, p-PI3K, AKT, p-AKT, BCL2 **(A)**, and BAX, Caspase 3, cl-Caspase 3 **(B)** in the presence or absence of the PI3K inhibitor LY294002. GAPDH was used as the loading control. **(C–G)** Quantitative analysis of the Western blot results: p-PI3K/PI3K ratio **(C)**, p-AKT/AKT ratio **(D)**, relative BCL2 protein level **(E)**, relative BAX protein level **(F)**, and cl-Caspase 3/Caspase 3 ratio **(G)**. **(H)** Representative immunofluorescence images of TUNEL staining in HepG2 cells. Red fluorescence indicates TUNEL-positive apoptotic cells, and blue fluorescence indicates DAPI-stained nuclei (Scale bar: 50 μm). **(I)** Quantitative analysis of the ratio of TUNEL-positive cells. Data are expressed as mean ± SD. *p < 0.05, **p < 0.01, ***p < 0.001, ****p < 0.0001.

Taken together, these *in vitro* results demonstrated that Lut protects hepatocytes against GCDCA-induced apoptosis by activating the PI3K/AKT signaling cascade, and that intact PI3K/AKT activity is indispensable for this anti-apoptotic function.

## 4 Discussion

Lut is the characteristic bioactive constituent and pharmacopoeial quality control marker of *S. chinensis* Benth. (Beiliujinu), a traditional herbal remedy long prescribed for damp-heat jaundice and hepatobiliary disorders. Both cellular and animal assays confirmed that Lut effectively attenuates cholestatic liver injury: it improved serum biochemical markers and hepatic histopathology in ANIT-treated mice, and protected HepG2 cells from GCDCA-induced apoptosis. To dissect the molecular pathways underlying this hepatoprotective effect, we performed network pharmacology analysis and transcriptomic screening of liver tissues. Both approaches converged on the PI3K/AKT signaling cascade and apoptosis as key regulatory targets. Subsequent experiments using TUNEL staining and Western blotting were carried out to verify whether Lut alleviated ANIT-induced cholestasis via the PI3K/AKT-dependent apoptotic cascade. WB results showed that ANIT markedly reduced the ratios of p-PI3K/PI3K and p-AKT/AKT, downregulated BCL2, and upregulated BAX and cl-Caspase 3. In contrast, Lut treatment reversed these abnormal protein expressions in either cell or animal experimental models. Furthermore, rescue assays using the PI3K inhibitor LY294002 validated the indispensable role of the PI3K/AKT pathway in Lut-mediated anti-apoptotic activity. SPR analysis demonstrated a direct binding interaction between Lut and PI3K in a concentration-dependent manner, and molecular docking further predicted that Lut occupied the ATP-binding pocket of the PI3K kinase domain and interacted with several surrounding residues. Collectively, these data demonstrate that Lut exerts anti-apoptotic effects via PI3K engagement and subsequent activation of the PI3K/AKT signaling pathway.

Based on our findings, we propose a refined mechanistic model for Lut’s hepatoprotective action against cholestasis. While the anti-inflammatory and antioxidant capacities of Lut have been well documented (Shanmugam et al., 2016; Yang et al., 2024; Cao et al., 2025), the present study is the first to identify its direct target-specific anti-apoptotic function in this disease context. Accumulated evidence has demonstrated that pharmacological reactivation of the PI3K/AKT pathway alleviates ANIT-induced cholestatic liver injury, although these studies have primarily focused on the anti-inflammatory and antioxidant consequences of pathway activation, without establishing a direct mechanistic link between PI3K/AKT signaling and the mitochondrial apoptotic execution machinery (Ma et al., 2015; Cao et al., 2023; Lyu et al., 2025; Wang et al., 2026). This observation is also supported by studies on small-molecule hepatoprotectants in bile acid-stimulated hepatocytes (Woudenberg-Vrenken et al., 2013). Consistent with previous reports, we found that Lut restored PI3K and AKT phosphorylation in ANIT-exposed mice and protected GCDCA-challenged HepG2 cells from apoptosis. More importantly, this study clarifies the downstream apoptotic execution mechanism. Serving as a master modulator of cellular survival, the PI3K/AKT signaling cascade governs the equilibrium among BCL2 family members. Correspondingly, Lut elevated BCL2 expression while reducing BAX levels to raise the BCL2/BAX ratio. This shift in protein equilibrium restricts permeabilization of the mitochondrial outer membrane, and curbs the activation of Caspase 3, the primary execution protease driving terminal apoptotic events. Our results therefore indicate that Lut blocks the intrinsic mitochondrial apoptotic cascade downstream of PI3K/AKT activation.

Notably, GCDCA, the major toxic bile acid driving cholestatic hepatocyte injury, is well-documented to suppress PI3K/AKT phosphorylation and initiate mitochondrial apoptosis (Hohenester et al., 2010; Tatsukawa et al., 2026), a mechanism we recapitulated in our *in vitro* model. In contrast, Lut directly engages the PI3K kinase domain and reverses this pathological suppression, recovering the survival signaling cascade disrupted by bile acid toxicity. This reciprocal regulation—suppression by an endogenous toxic metabolite and restoration by a natural flavonoid—highlights a targeted corrective mechanism that goes beyond the general radical-scavenging activity commonly attributed to polyphenolic compounds. Our molecular docking results reveal that Lut fits into the conserved ATP-binding pocket of the PI3K kinase domain and forms stable hydrogen bonds with multiple pocket-lining residues (Figure 4B), adopting a binding pose spatially similar to the well-known PI3K inhibitor LY294002 (Walker et al., 2000). Nevertheless, this docking-predicted pocket occupancy does not constitute biochemical proof of ATP-competitive inhibition. Unlike LY294002, a pan-PI3K inhibitor that robustly suppresses PI3K kinase activity, Lut exerts a pathway-restoring effect under cholestatic stress, in which PI3K/AKT signaling is repressed by toxic bile acids. We hypothesize that Lut binding within the ATP-binding pocket may sterically counteract bile-acid-triggered inhibitory conformational rearrangement of PI3K, thereby restoring basal kinase activity, rather than driving complete inhibition or excessive pathway hyperactivation. This hypothetical model aligns with previously-reported context-dependent regulatory properties of Lut toward the PI3K/AKT pathway: Lut suppresses PI3K/AKT signaling in various tumorigenic cell lines (Ma et al., 2015; Kang et al., 2017), whereas it rescues impaired PI3K/AKT activity under stress-induced hepatic injury, including drug-provoked hepatotoxicity and NAFLD (Wang et al., 2021; Hong et al., 2022). Such bidirectional modulation implies that the functional output of Lut-PI3K interaction is largely determined by the intrinsic basal activity status of the PI3K/AKT cascade within a given pathological microenvironment.

In cholestasis, hepatocyte apoptosis does more than simply eliminate damaged cells—it actively fuels disease progression. Dying hepatocytes release DAMPs that trigger Kupffer cell activation and monocyte recruitment, setting off a cycle of sterile inflammation that, over time, drives hepatic stellate cell transdifferentiation, ductular reaction, and periportal fibrosis (Schwabe and Luedde, 2018; An et al., 2020; Kisseleva and Brenner, 2021). By blocking mitochondrial apoptosis at the point where toxic bile acids initiate this cascade, Lut may indirectly limit the downstream inflammatory and fibrotic responses that sustain cholestatic liver injury. Although direct evidence for this secondary benefit requires validation in chronic fibrosis models, the established mechanistic coupling between sustained hepatocyte apoptosis and fibrogenesis provides a rational basis for this hypothesis.

Beyond anti-apoptotic activity, Lut also restored the expression of bile acid transporters and reduced inflammatory cytokine levels *in vivo*, reflecting its multifunctional pharmacological properties. Notably, *in vitro* rescue experiments showed that LY294002 not only abolished Lut’s anti-apoptotic effect but also impaired its antioxidant capacity (i.e., it abrogated Lut-mediated increases in GSH and SOD, as well as Lut-driven reduction in MDA). This suggests that the antioxidant effect of Lut is partially dependent on PI3K/AKT activation, rather than merely resulting from its intrinsic free-radical-scavenging activity. Importantly, these observations indicate that the anti-apoptotic and antioxidant actions of Lut are not two fully independent parallel effects under cholestatic stress; instead, they operate in a sequential, hierarchical manner. We speculate that direct binding of Lut to PI3K initiates a coordinated protective network. In this hierarchical model, target-driven PI3K/AKT pro-survival signaling preserves mitochondrial function and curtails ROS overproduction, thereby mediating both anti-apoptotic and secondary antioxidant hepatoprotection. Nevertheless, the causal contribution of PI3K to Lut-regulated bile acid transporters and anti-inflammatory responses cannot be established based on current data. Given extensive crosstalk between the PI3K/AKT pathway and the NF-κB, FXR, and Nrf2 axes (Yang et al., 2024; Cao et al., 2025), whether Lut’s effects on bile acid transport and inflammation are directly triggered by PI3K activation remains an open question and requires dedicated experimental validation.

Beyond these functional observations, our findings are directly relevant to a prior report by Sun et al., who used DARTS combined with molecular docking to propose an interaction between Lut and PI3K in an oxidative stress model of non-alcoholic fatty liver disease (Sun et al., 2020). While that work provided an important initial clue, three gaps remained: it did not quantify the binding affinity, it lacked functional rescue experiments to establish pathway dependency, and it did not examine whether this interaction operated in cholestatic liver injury. The present study addresses each of these gaps: we provide SPR-based quantitative binding data, employ LY294002 rescue assays to confirm that PI3K/AKT activity is functionally required for Lut’s anti-apoptotic effect, and extend the Lut–PI3K interaction into cholestatic liver injury for the first time. The convergence between independent target engagement methods—DARTS and SPR—across distinct disease contexts strengthens the case for PI3K as a specific molecular target of Lut.

A notable discrepancy was observed between the kinetic and steady-state KD values derived from the SPR analysis (kinetic KD = 9.036 × 10^−5^ M; steady-state KD = 2.82 × 10^−4^ M, a >3-fold difference). Such divergence is commonly encountered for small-molecule interactions of moderate affinity, where equilibrium responses at lower concentrations may be influenced by minor bulk-shift effects or low-level non-specific binding, leading to overestimation of the steady-state KD (Myszka et al., 1998; Giannetti et al., 2008; Rich and Myszka, 2010). Given that the kinetic analysis draws on the full time-course of association and dissociation phases and fits the 1:1 Langmuir model with a minimal Chi^2^ value (2.03 RU^2^), the kinetic-derived KD is considered the more accurate representation of the Lut–PI3K binding affinity. The steady-state KD is reported for data completeness and transparency, but the kinetic value is used as the primary affinity parameter throughout this study.

As polyhydroxy flavonoids inherently carry high risks of pan-assay interference (PAINS) (Baell and Holloway, 2010), reliably distinguishing genuine target binding from non-specific off-target interactions remains a major challenge for mechanistic investigations of this compound class. To tackle this issue systematically, we adopted SPR as a quantitative biophysical detection platform, in which all sensorgrams were double-corrected with reference channel subtraction and blank buffer baseline deduction to eliminate background noise. Lut yielded concentration-dependent, saturable binding signals reaching a distinct plateau near the theoretical Rmax and fitted excellently to a standard 1:1 Langmuir model with minimal Chi^2^ deviation. This saturable, reversible binding pattern is fundamentally incompatible with the unbounded, drifting sensorgrams typical of aggregatory PAINS compounds, providing a clear biophysical distinction between specific target engagement and non-specific adsorption (Giannetti et al., 2008). Complementing the SPR data, molecular docking predicts that Lut occupies the conserved ATP-binding pocket within the PI3K kinase domain and forms hydrogen-bond contacts with multiple pocket-lining residues (Figure 4B), a binding mode consistent with the known ability of flavonoid scaffolds to occupy kinase ATP sites (Walker et al., 2000; Hou and Kumamoto, 2010). Importantly, pharmacological blockade of PI3K with LY294002 nearly abolished all Lut-mediated protective effects in cellular models, confirming that its bioactivity depends on intact PI3K function. Together, the convergence of quantitative SPR kinetics, structural docking, and pathway-specific functional rescue supports specific molecular recognition that cannot be explained by non-selective polyphenol interference.

Our SPR measurement yielded a kinetic KD of 9.036 × 10^−5^ M for the Lut-PI3K interaction, representing mid-micromolar affinity consistent with reported values for natural flavonoid-kinase interactions (Walker et al., 2000). This moderate affinity likely reflects the inherent structural characteristics of the flavonoid scaffold relative to optimized synthetic PI3K inhibitors, which typically achieve nanomolar potency. We propose that this moderate-affinity binding underpins the context-dependent regulatory feature of Lut-PI3K interaction. In contrast to high-potency synthetic PI3K inhibitors that exert constitutive kinase suppression, Lut is capable of rescuing suppressed PI3K/AKT signaling under cholestatic bile-acid-mediated stress, without inducing aberrant over-activation under basal conditions. This context-dependent activation, enabled by moderate-affinity binding, aligns with the bidirectional regulatory behavior of Lut described above.

Among flavonoids investigated in cholestasis models, kaempferol and quercetin have been reported to activate PI3K/Akt signaling (You et al., 2023; Li et al., 2026), although those findings relied on Western blot and inhibitor-based evidence without direct biophysical confirmation of target engagement. Other flavonoids, such as apigenin, have been studied mainly for their regulation of FXR-dependent bile acid transport or inflammatory pathways, with limited attention to the mitochondrial apoptotic cascade (Zheng et al., 2021; Liu et al., 2024). Our study adds to this picture by providing quantitative SPR data that validate direct Lut–PI3K binding, resolving the downstream PI3K/BCL2/ Caspase 3 anti-apoptotic signaling axis, and linking this mechanism to the traditional application of *S. chinensis*. Unlike these flavonoids, whose protective effects have been primarily characterized at the pathway level without a validated direct molecular target, Lut acts through a defined molecular target (PI3K) and a specific downstream execution pathway (mitochondrial apoptosis). This target-specific, apoptosis-directed mechanism distinguishes Lut from other flavonoids in the cholestatic context and provides a clear molecular rationale for its development as a targeted adjunctive therapy.

Lut possesses several properties that make it an attractive candidate for adjuvant therapy in cholestasis. Synthetic PI3K inhibitors in clinical use carry well-documented risks of on-target and off-target toxicities (Greenwell et al., 2017); Lut, in contrast, is a dietary flavonoid with a long history of safe human consumption and a favorable profile for prolonged oral administration. Current first-line therapy with UDCA fails to achieve adequate biochemical response in approximately 40% of patients, and the second-line agent OCA is limited by pruritus and metabolic side effects (Floreani and Mangini, 2018). UDCA acts primarily by modifying the bile acid pool through FXR, whereas Lut targets the upstream PI3K survival pathway, providing anti-apoptotic, antioxidant, and anti-inflammatory protection that complements the mechanism of UDCA rather than overlapping with it. In our mouse model, Lut was effective at oral doses of 30 and 60 mg/kg, offering a starting point for dose optimization in further preclinical work.

These mechanistic findings are directly relevant to understanding the traditional therapeutic application of the source herb. *Siphonostegia chinensis* Benth. (Beiliujinu) has been recorded in classical materia medica for treating damp-heat jaundice, a syndrome that overlaps clinically with cholestatic liver disorders. As its official pharmacopoeia marker, Lut directly targets PI3K to activate anti-apoptotic signaling, thereby protecting hepatocytes from bile acid toxicity. This defined molecular mechanism provides a plausible pharmacological basis for the traditional application of Beiliujinu in cholestatic conditions.

Several limitations should be noted. The principal limitation related to our core novel claim—direct PI3K targeting of Lut in cholestatic hepatocytes—originates from the cell-free nature of our SPR binding assays. Although we obtained robust, quantitative kinetic and steady-state KD values confirming Lut–PI3K interaction in purified protein systems, these cell-free data alone cannot definitively verify that Lut engages PI3K at physiologically achievable intracellular concentrations within intact bile acid-stressed hepatocytes (HepG2). A previously published DARTS assay (Sun et al., 2020) independently validated Lut–PI3K intracellular binding in BRL-3A liver cells; however, that work used a non-cholestatic NAFLD experimental context with a distinct cell line, so its supporting evidence cannot fully substitute for cellular target validation in our cholestasis model. To strengthen the translational relevance of our SPR results, follow-up work will conduct cellular thermal shift assay (CETSA) in GCDCA-challenged HepG2 cells to directly confirm intracellular Lut–PI3K target engagement under our disease-specific conditions (Martinez Molina et al., 2013). Furthermore, genetic validation using PI3K knockdown or knockout models would provide more definitive causal evidence linking PI3K to Lut-mediated hepatoprotection in cholestatic hepatocytes, which represents another important direction for future work.

At the molecular level, Lut bound PI3K with mid-micromolar affinity, which is typical of many natural products but also implies that structural optimization will be needed to improve druggability. SPR and molecular docking placed the binding within the kinase domain, yet the conformational changes induced by Lut remain unclear; protein co-crystallization and site-directed mutagenesis will be necessary to resolve them. In addition, the preferential binding capacity of Lut across distinct class I PI3K isoforms is yet to be characterized. Meanwhile, Lut’s intrinsic antioxidant activity may also participate in PI3K/AKT regulation. *In vitro* experiments were solely conducted on HepG2 cells, a hepatoma-derived cell line that, while widely used for studying hepatocyte apoptosis, differs from normal hepatocytes in the expression of drug-metabolizing enzymes and bile acid transporters (Wilkening et al., 2003). Validation of the key anti-apoptotic phenotypes in primary hepatocytes would enhance the translational relevance of these findings. For *in vivo* research, this study adopted an acute ANIT model, and long-term effects on bile duct proliferation and liver fibrosis need to be verified in chronic models such as DDC-induced cholestasis.

Additionally, as a flavonoid aglycone, Lut exhibits limited aqueous solubility and undergoes extensive first-pass glucuronidation and sulfation in the intestine and liver, resulting in low systemic bioavailability of the parent compound (Chen et al., 2007; Di Lorenzo et al., 2021). Whether the *in vivo* efficacy observed at 30–60 mg/kg oral doses is mediated by Lut itself, its circulating conjugated metabolites, or a combination thereof remains to be clarified through pharmacokinetic-pharmacodynamic correlation studies. Furthermore, although our data establish PI3K as a critical target, the multi-target nature of flavonoids suggests that Lut may concurrently engage additional signaling nodes that contribute to its overall hepatoprotective profile. Systematic chemoproteomic profiling would help delineate the complete target landscape and inform rational structural optimization. Exploring the combined application of Lut and UDCA is also a valuable direction for follow-up research. These issues will be addressed in our subsequent work.

## 5 Conclusion

Taken together, our findings demonstrate that Lut directly binds to PI3K and activates the PI3K/AKT anti-apoptotic signaling axis, thereby protecting hepatocytes against ANIT-induced cholestatic injury (Figure 8). Lut is the primary bioactive flavonoid of *S. chinensis* Benth. (Beiliujinu), a classic herbal medicine historically indicated for damp-heat jaundice. By elucidating this PI3K-directed anti-apoptotic mechanism, our data provide a mechanistic basis for this traditional ethnomedicinal application. Beyond its relevance to Beiliujinu, Lut is also widely distributed in edible plants as a dietary flavonoid with an established safety profile. It therefore warrants further evaluation as a candidate adjuvant to UDCA-based therapy, particularly for the substantial proportion of cholestatic patients who respond inadequately to UDCA alone. Future studies incorporating chronic cholestasis models and UDCA combination regimens will be required to advance these preclinical observations toward clinical translation.

**FIGURE 8:**
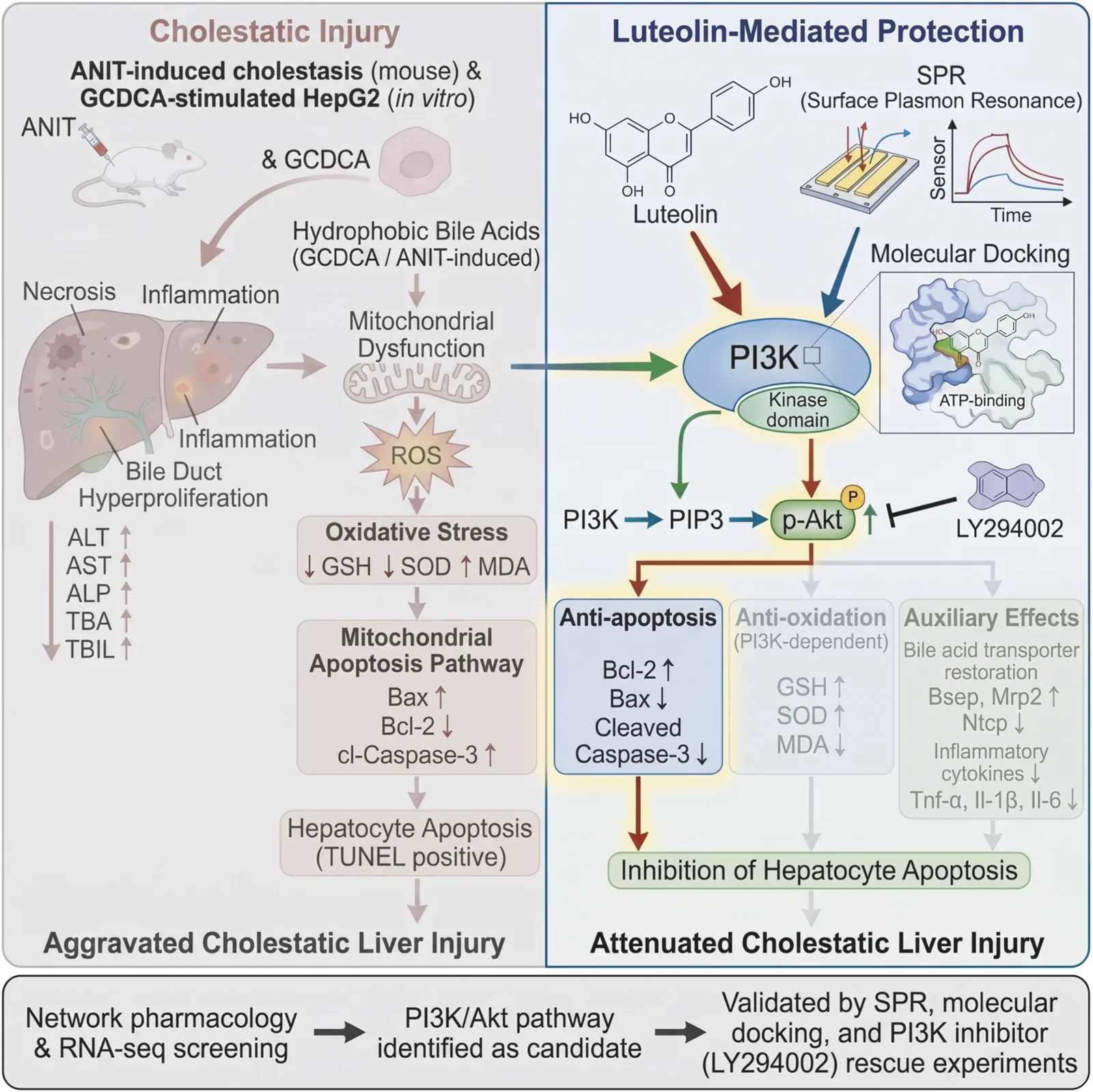
Schematic diagram of the protective mechanism: Lut alleviates ANIT-induced cholestatic liver injury through PI3K/AKT-dependent inhibition of hepatocyte apoptosis.

## Data Availability

The raw RNA-seq transcriptome data reported in this paper have been deposited in the Genome Sequence Archive (GSA) of the National Genomics Data Center, China National Center for Bioinformation, accession number CRA050800. All quantitative experimental raw data (serum biochemistry, qPCR, Western-blot quantification, and histological-related data) supporting the findings have been deposited in the OMIX repository under accession number OMIX021295.
